# Supplementary figures and images for: Fat mass loss correlates with faster disease progression in amyotrophic lateral sclerosis patients: Exploring the utility of dual-energy x-ray absorptiometry in a prospective study
Source: PLoS One. 2021 May 6;16(5):e0251087. doi: 10.1371/journal.pone.0251087 (PMC8101939; doi:10.1371/journal.pone.0251087)

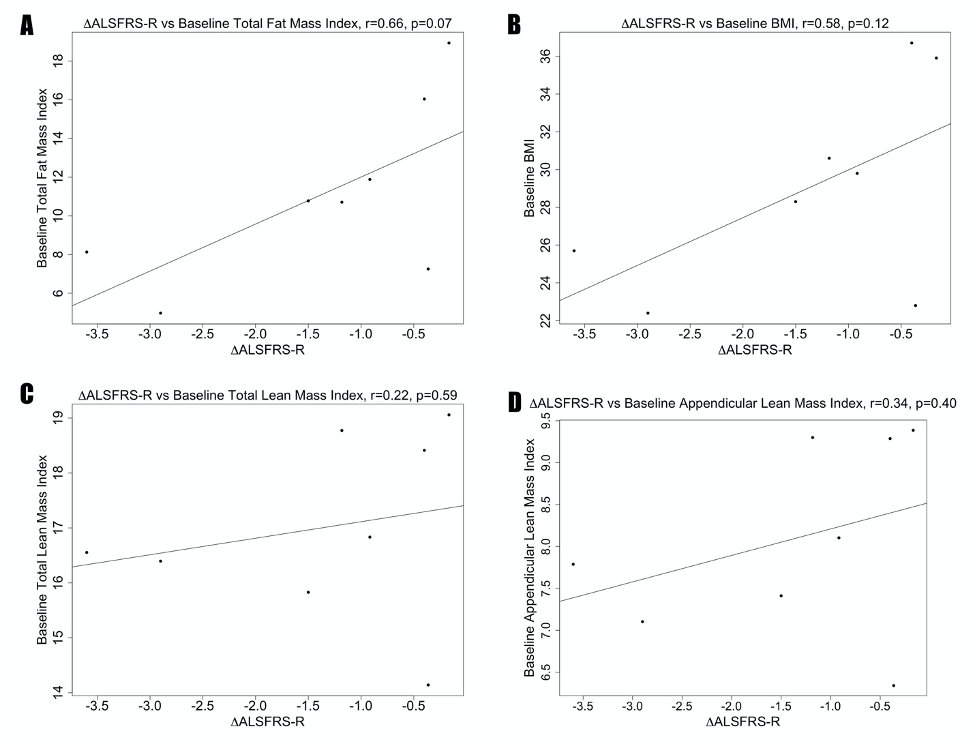

Supplement: S1 Fig — ΔALSFRS-R and (A) Baseline Total Fat Mass Index, (B) Baseline BMI, (C) Baseline Total Lean Mass Index and (D) Baseline Appendicular Lean Mass Index are not significant. (TIF) [file pone.0251087.s001.tif]
